# Personalized Alzheimer's disease risk profiling in healthy middle‐aged individuals: The Cedars‐Sinai Memory & Healthy Aging Program

**DOI:** 10.1002/alz70857_101637

**Published:** 2025-12-25

**Authors:** Zaldy S Tan, Nabeel Qureshi, Andrew Hirsch, Stephanie Bray, Mitzi M. Gonzales, Celina Shirazipour, Hugo J. Aparicio, Fatemeh Ramezani, Sarah Kremen, Nancy Sicotte

**Affiliations:** ^1^ Cedars‐Sinai Medical Center, Los Angeles, CA, USA; ^2^ RAND Corporation, Santa Monica, CA, USA; ^3^ Boston University School of Medicine, Boston, MA, USA; ^4^ Department of Neurology, Boston University Chobanian & Avedisian School of Medicine, Boston, MA, USA

## Abstract

**Background:**

Addressing modifiable medical and lifestyle risk factors may have the potential to reduce incidence of Alzheimer's disease and related dementias (ADRD). The Cedars‐Sinai Memory & Health Aging Program (MHAP) is a clinical and research program that promotes brain health through personalized risk profiling and risk reduction among at risk, asymptomatic adults. In this study, we describe the demographics and risk profiles of participants seen in the first year of the program.

**Method:**

All Cedars‐Sinai Medical Center patients received an email message and a link to the MHAP website. Eligibility criteria included 1) Age 40+ years; 2) absence of a cognitive or neurological diagnosis; 3) 2 risk factors for ADRD (only applicable to adults <65 years). Participants were asked to complete a pre‐visit questionnaire on family and medical history, and validated surveys assessing ADRD risk factors identified by the Lancet Commission. Data analysis included descriptive statistics of participant demographics and survey responses.

**Result:**

The mean age of the cohort (*N* = 64) was 59.5 years (range: 40‐87). Most patients were female (59.4%), non‐Hispanic white (75%) or Asian (9.4%), and highly educated (95.3% college graduate or higher). More than half had the following ADRD risk factors: sleep disorder (75%), family history of ADRD in a first degree relative (69%), low physical activity (61%), low levels of socialization (55%), obstructive sleep apnea (55%), elevated LDL/Cholesterol (53%), and low MIND diet score (52%). Between a third and half of patients had the following additional risk factors: high blood pressure (42%), history of traumatic brain injury (TBI) (36%), and depressed mood (33%). Fifty‐one participants completed the Montreal Cognitive Assessment (MoCA) and the average score for this highly educated cohort was 26.1 (range: 15‐30), with difficulties on items assessing delayed recall (86%), language (33%), visuospatial/executive (29%), orientation (28%), and attention (26%).

**Conclusion:**

Personalized risk profiling for ADRD risk showed high rates of modifiable ADRD risk factors, including sleep disorder, elevated LDL cholesterol, low MIND diet scores, and low levels of physical activity and socialization. Future research will examine the feasibility and effectiveness of ADRD risk mitigation in measures of cognitive and structural brain aging.

